# Inner ear ossification and mineralization kinetics in human embryonic development - microtomographic and histomorphological study

**DOI:** 10.1038/s41598-017-05151-0

**Published:** 2017-07-06

**Authors:** Céline Richard, Guillaume Courbon, Norbert Laroche, Jean Michel Prades, Laurence Vico, Luc Malaval

**Affiliations:** 1 ENT, Head and Neck Surgery Department, Lausanne University Hospital (CHUV), Lausanne, Switzerland; 2 The Laboratory for Investigative Neurophysiology (The LINE), Department of Clinical Neurosciences, University Hospital Center and University of Lausanne, Lausanne, Switzerland; 3 Laboratoire de Biologie des Tissus Ostéoarticulaires, INSERM, U1059 Sainbiose, Université de Lyon, Université Jean Monnet, Faculté de Médecine, Campus Santé Innovation, 42023 Saint-Étienne, France; 4 ENT, Head and Neck Surgery Department, Université de Lyon, Université Jean Monnet, CHU, Hôpital Nord, 42055 Saint-Étienne, France

## Abstract

Little is known about middle and inner ear development during the second and third parts of human fetal life. Using ultra-high resolution Microcomputed Tomography coupled with bone histology, we performed the first quantitative middle and inner ear ossification/mineralization evaluation of fetuses between 17 and 39 weeks of gestational age. We show distinct ossification paces between ossicles, with a belated development of the stapes. A complete cochlear bony covering is observed within the time-frame of the onset of hearing, whereas distinct time courses of ossification for semicircular canal envelopes are observed in relation to the start of vestibular functions. The study evidences a spatio-temporal relationship between middle and inner ear structure development and the onset of hearing and balance, critical senses for the fetal adaptation to birth.

## Introduction

Hearing is a complex multilevel process. The first auditory step is performed by three compartments, the external, middle and inner ears, located inside the temporal bones, bilateral osseous structures situated on each side of the base of the skull. In mammals, sound vibrates the tympanic membrane and relays through the three middle ear ossicles to the inner ear whose characteristics in human allow responses to sound frequencies from 20 Hz up to 20 kHz. Inner ear structures are involved in both hearing and balance functions with the cochlea and the vestibule, respectively situated in the antero-inferior and posterior-superior parts of the labyrinth. The three ossicles, malleus, incus and stapes, and their articulations (incudomalleal and incudostapedial) exhibit specific morphological and histological properties required to optimize sound transduction (conversion of pressure waves into oscillating displacements) and transmission, amplification of the signal by a lever action and matching of the acoustic air impedance with the higher cochlear liquid impedance in order to deliver high energy vibrations to the fluid–filled inner ear structures.

The auditory signal from the middle ear is delivered via the stapes to the fluid-filled membranous labyrinth of the spiral organ of hearing, known as the Organ of Corti, located within the cochlear duct and whose hair cells distributed along the length of the cochlear basilar membrane convert and encode vibrations into electroneural stimulations through the auditory nerve to the auditory system. On the posterior part of the membranous labyrinth, the inner ear balance system consists of three semicircular canals (SCC), functioning as gyroscopes which detect rotations, and two gravity receptors, the utricule and saccule (otolith system) which respond to both linear acceleration and gravity. These structures allow the vestibule to act as a high sensitive sensor, which detects head movements along any axis, and serves three main functions: (1) the control of spinal reflexes involved in posture adjustment, (2) the control of eye movements, (3) the perception of motions and spatial orientation. The inner ear balance system sends projections to the vestibular nuclei and different structures of the brainstem^1, 2^. Inner ear fluid vibrations stimulate auditory and motion receptors, remarkably effective mechanosensors for both hearing and balance which provide subcortical and cortical structures with precise and accurate inputs.

Although the middle and inner ear osseous structures, similar to the ethmoid or sphenoid bones for instance are embedded in the skull base, they exhibit peculiar developmental features and ossification patterns that differ from the other part of the cranial base^3^. Different patterns of inner ear developments have been suggested, some advocating a prenatal maturation of the bony labyrinth based on the observation of complete ossification of the surrounding otic capsule by seven months in utero^4^, and others claiming that the temporal bone undergoes further postnatal changes^5^. However, in most previous works, investigations of fetal inner and middle ear ossification in human fetuses were based on histological examination of decalcified temporal bones specimens^6^ and/or on three-dimensional (3D) images extrapolated from serial histological slices^7^, which made accurate measurements quite challenging.

Notwithstanding the development of modern imaging technologies such as Computed Tomography or Magnetic Resonance Imaging and their use in temporal bone research^8, 9^ the assessment of bone development remains complex. Such technologies cannot provide a resolution under 0.5 mm and do not allow the evaluation of bone mineral deposition. Microcomputed Tomography (*μ*CT) on the other hand, provides both high resolution 3D images of structures -as opposed to the degraded spatial resolution of histological reconstructions^10^- and at least semi-quantitative assessment of bone mineralization. Additionally, the development in the bone and skeletal research field of histological and histomorphometric techniques for imaging in praiseworthy morphological details^11^ and analyzing quantitatively^12^ undecalcified specimens has allowed for years the direct evaluation of human bone structure, development and remodeling. Such detailed analysis is of direct relevance to the fields of audiology and otologic surgery in order to develop accurate diagnosis, better operation and care strategies. In order to provide a more comprehensive information of human ear development, we describe in the present report the use of two complementary techniques so far never integrated for this purpose, namely *μ*CT and undecalcified histomorphometry for the spatio-temporal evaluation of both middle and inner ear ossification and mineralization in fetuses from 17 to 39 weeks (wks) of gestational age.

## Results

**Figure 1:**
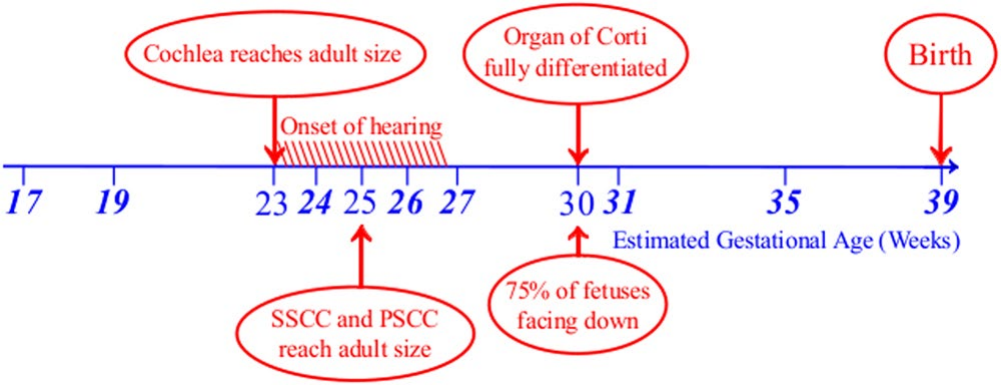
Estimated gestational age of collected specimens (bold italic) within the time-course of inner ear embryonic development.

Figure 1 summarises the time-course and major events of embryonic ear development along with the specimen collection schedule for this study.

### Middle ear

**Figure 2:**
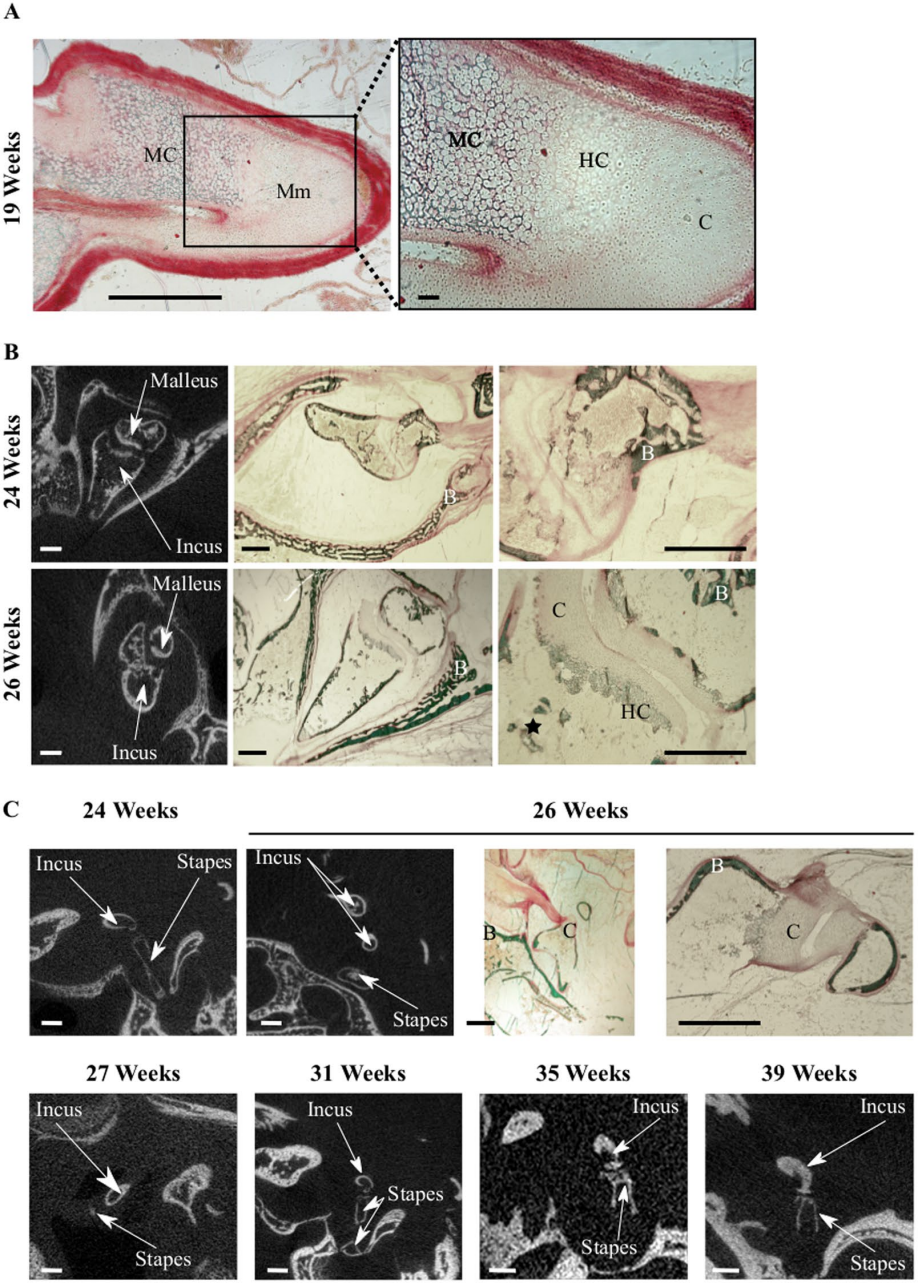
Inner ear development – Inter ossicle articulations. (**A**) Goldner stained sections of the Malleus at 19 weeks. Mm: Manubrium, B: Bone, C: Cartilage, HC: Hypertrophic Cartilage, MC: Mineralized Cartilage. 2D *μ*CT images and Goldner stained sections of the incudomallear (**B**) and incudostapedial (**C**) articulations between 24 and 39 weeks. Bars = 1.0 m, except inset in A: 100 *μ*m.

**Figure 3:**
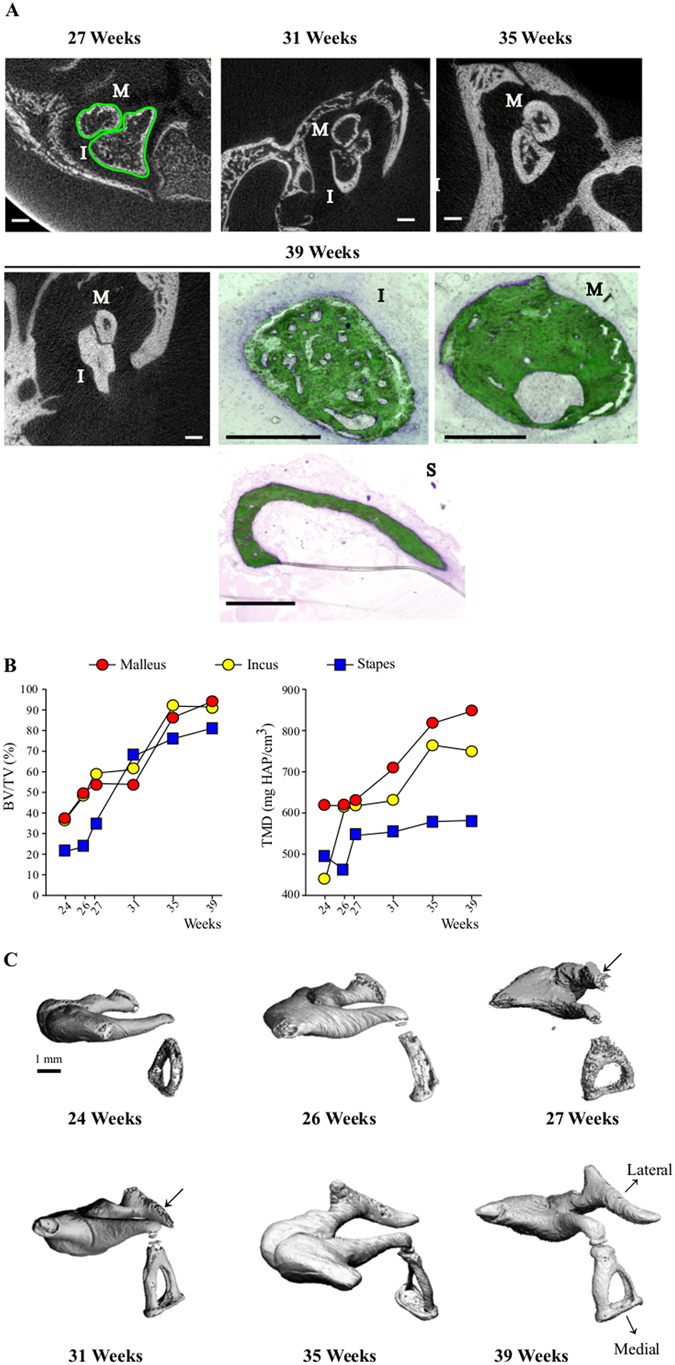
Inner ear development-Ossicle bone maturation. (**A**) 2D *μ*CT and Goldner stained sections of ossicles at 27, 31, 35 and 39 weeks. M: Malleus, I: Incus, S: Stapes. White bars = 1.0 mm, black bars = 0.5 mm. ROI definition for *μ*CT analysis and 3D rendering are outlined in green in the upper left image. (**B**) Time-course of bone volume (BV/TV) and tissue mineral density (TMD) increase and (**C**) 3D *μ*CT rendering of ossicles in successive weeks of gestation: the lateral and medial orientation of the ossicular chain are indicated, thick arrows = manubrium.

We detected no mineralized middle ear structure by *μ*CT at 17 and 19 weeks (wks), but the presence of mineralized cartilage in the anlage of the malleus manubrium was visible at 19 wks in Goldner stained sections (Fig. 2A). At 24 wks, all 3 ossicles appeared ossified, with thin bone cortices and large marrow cavities, where a few bone trabeculae could be observed, some still in part cartilaginous (Fig. 2B). At this stage, unossified cartilage was visible only in the contact zones between ossicles, as premises of the articular cartilage (Fig. 2B). The incudomallear articulation shows diarthrosis characteristics with hyaline cartilage on articular surfaces at 26 wks and an ossified inner layer visible on microCT (Fig. 2B and C). The mushroom-shaped lenticular process of the incus can be divided into two parts: a stem and a cap zone whose ossification is progressive (Fig. 3C). The mineralized cortex of the cap is visible on the 26 wks specimen (Figs 2C and 3C) whereas complete ossification of both the stem and the cap appears at 35 wks (microCT, Fig. 3C). The mineralization of the corresponding articulating surface of the head of the stapes is visible at 31 wks with a remaining cartilage zone between the articulating surface and the neck of the stapes (Fig. 3C), this cartilaginous area is mineralized on the 35 wks specimen (Figs 2C and 3C). Joints between the ossicles are diarthrodial^13^ and ossicule muscles are peculiar in that their enthesis contain elastic fibers^14^. The incus-stapes articulation (Fig. 2C) progressively appears in tomographic images, and is fully shaped only at 35 wks (Fig. 2C, see also Fig. 3C).

During embryonic development the thin cortices of the ossicles progressively thicken, obliterating the marrow cavity (Fig. 3A), whose remnants are still visible at 39 wks (Fig. 3A, bottom row). Quantification of bone volume in all 3 ossicles reflects this rapid filling-up with compact mineralized matrix observed between 27 and 35 wks (Fig. 3B). The delayed development of the stapes is visible in its lower BV/TV at most time-points, as well as in the stalled Tissue Mineral Density (TMD) of this ossicle throughout most development stages (Fig. 3B). The 3D images of the ossicles in connection confirm the slower ossification of the stapes and the late development of the incus-stapes articulation. They also reveal the much later ossification of the manubrium part of the malleus, whose mineralization is detected only at 27 wks, and complete at 31 wks (Fig. 3C, arrows).

### Cochlea

The inner ear consists of a set of interconnected soft tissue sacs and ducts known as “the membranous labyrinth” within interconnected bony spaces called “the bony labyrinth” or “otic capsule”, located in the petrosal bone. The bony cochlea is coiled around the modiolus, a central axis housing the cochlear branch of the auditory nerve. The tip of the cochlea is known as the apex and the basal cochlear part, called the base, ends near the oval window receiving the footplate.

**Figure 4:**
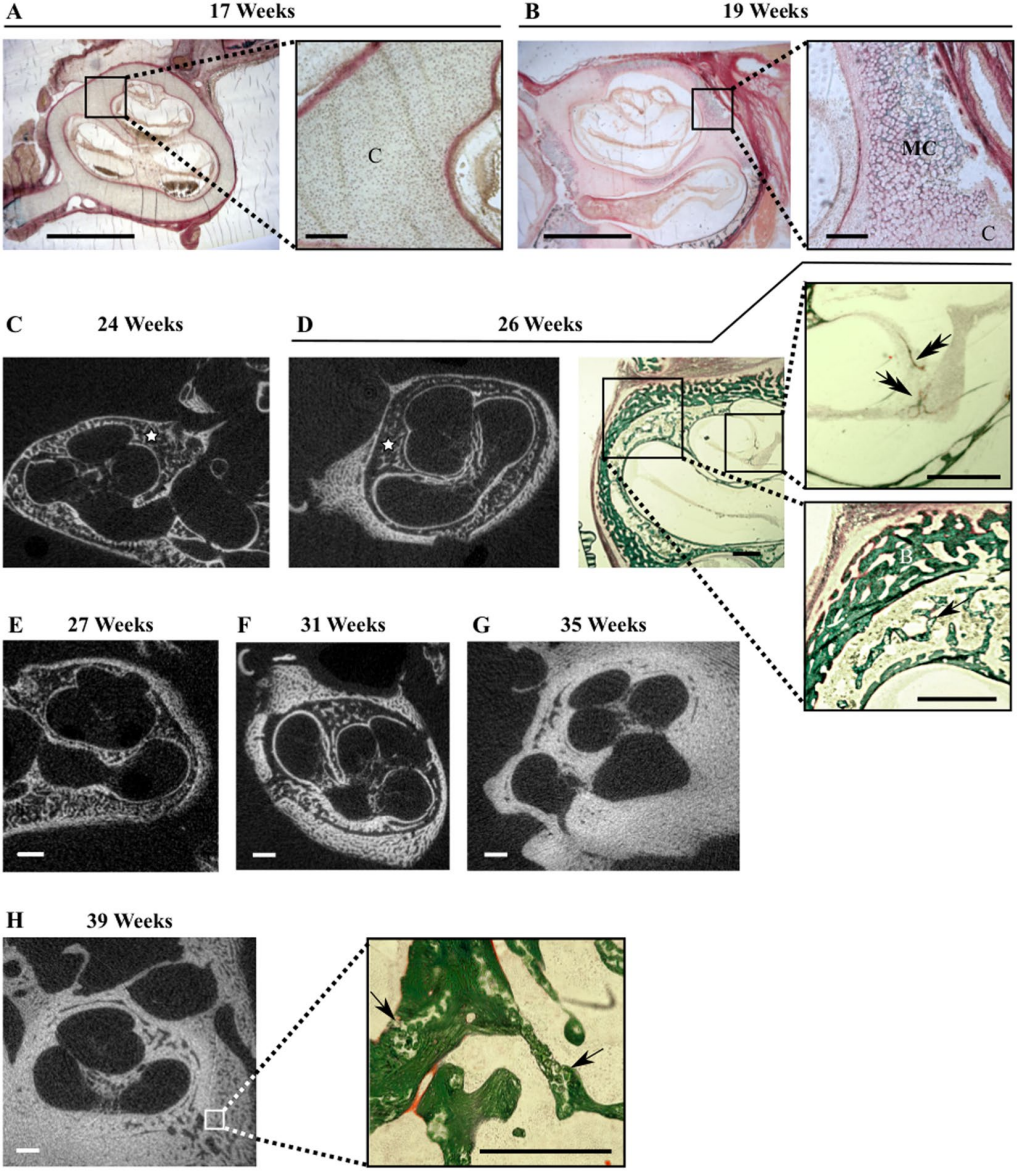
Ossification of the cochlear otic capsule. Goldner stained (**A**,**B** and **D**) and/or *μ*CT sections (**C**–**H**) of the cochlea between 17 and 39 weeks of gestation. In A: C = Cartilage, MC = Mineralized Cartilage, Stars in C and D: Trabecular area (“Middle Layer”), Simple arrows in D and H: Cartilage remains (clear areas), Double arrow in D: Membranous modiolar bone profiles. Bars = 1.0 mm, exc3ept for insets in A and B: 100 *μ*m.

**Figure 5:**
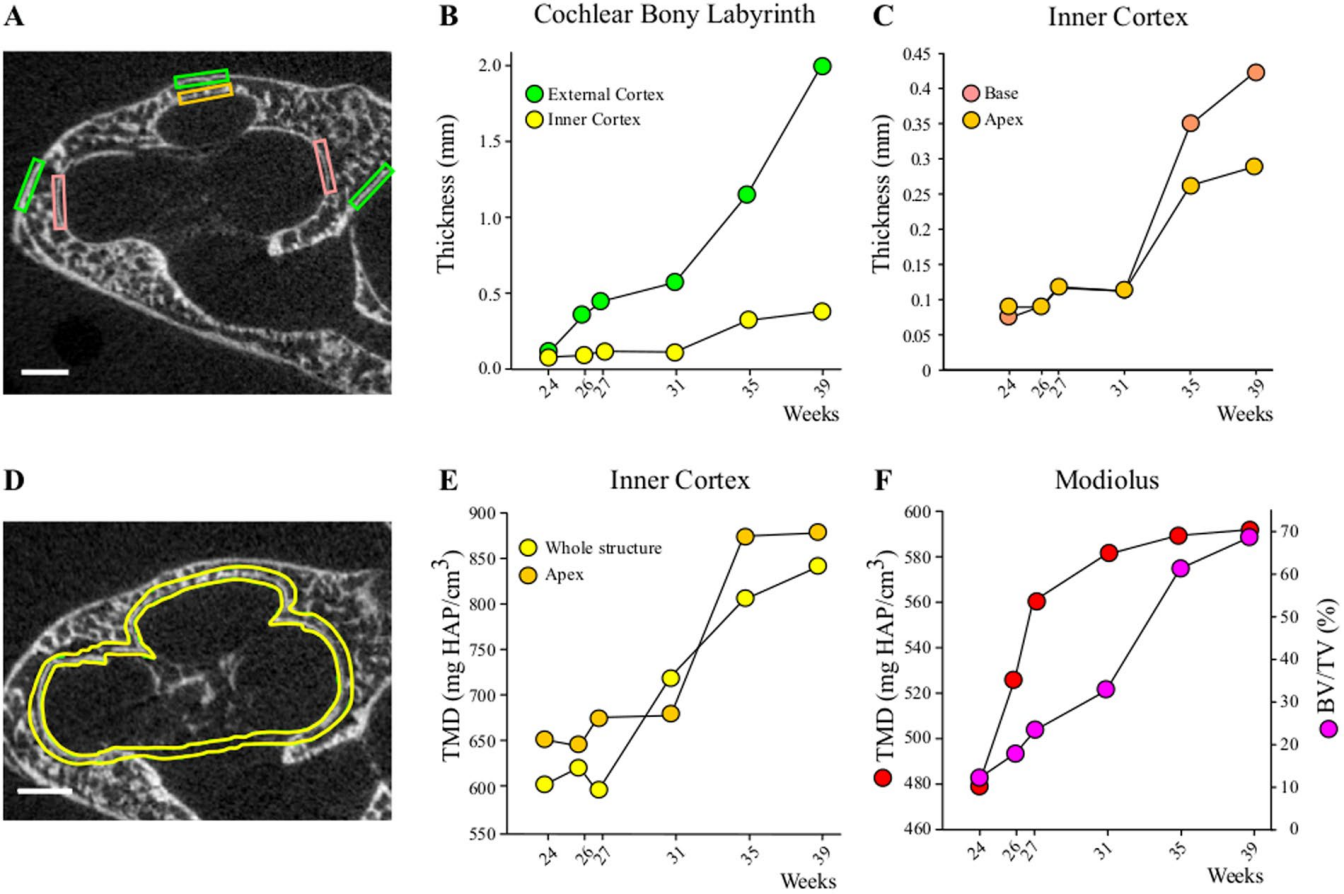
Time-course of cortical thickening and mineralization in the cochlear otic capsule. (**A**) The boxes locate the sites of thickness measurement of the outer (green boxes) and inner (orange apical box, pink basal boxes) cortical bones on a *μ*CT section at 24 weeks. Bar = 1 mm. Progressive thickening of (**B**) the external and internal cortices and (**C**) the basal and apical region of the internal cortical bone at successive weeks of gestation. (**D**) ROI definition for *μ*CT analysis of the inner cortical bone (yellow lines) on a *μ*CT section at 26 weeks. Time-courses of tissue mineral density (TMD) increase (**E**) in the apical (ROI = apical orange box in **A**) and the whole inner cortical bone (ROI in **D**) and of the ossification (BV/TV) and TMD increase in the modiolus (**F**, see Methods for ROI), at successive weeks of gestation.

At 17 wks, the cochlea is still surrounded by cartilage with no mineralized focus detected, either on the histological sections (Fig. 4A) or on *μ*CT images (not shown). Although still not detected by *μ*CT in our scanning conditions, merging foci of mineralized cartilage in the cochlear envelope are visible at 19 wks in Goldner sections (Fig. 4B). The ossification of the cochlear part of the otic capsule progresses throughout the development stages (Fig. 4C–H). Similar to middle ear ossicles, by 24 wks two individualized cortices delimit an area of bone marrow with trabeculae (Fig. 4C). These are still not fully ossified and include cartilage foci (Fig. 4D, lower inset, simple arrow), which are still present at birth (Fig. 4H, inset, arrows). During the fetal period, the otic capsule follows an outward-inward developmental pattern with a progressive thickening of the highly porous external cortex while the inner cortical bone remains thinner, as seen on histological sections (Fig. 4D) and *μ*CT images (Fig. 4C–H) and confirmed by measurements at specific basal and apical locations (Fig. 5B). The pace of outer cortical bone expansion is similar at the cochlear base and the apex until 31 wks, when the basal bone accelerates its thickening (Fig. 5C). Simultaneously a reduction in cortical bone porosity (Fig. 4G and H) and an increase of the tissue mineral density (TMD) are observed (Fig. 5E), reflecting the maturation of the bone matrix between 27 and 35 wks. The apical inner cortical layer of the cochlear otic capsule exhibits a higher degree of mineralization than the whole cochlea at all time points except 31 wks. The mineralization of the apex reaches its maximum around 35 wks, contrasting with the values for the whole structure which are still increasing at birth time (Fig. 5E). While the porosity of the outer and inner cortical bones of the cochlea decreases, their expansion progressively fills up the marrow areas, and only thin empty spaces remain at late stages (Fig. 4G and H, 35 and 39 wks). In accordance with a membranous ossification process^15^, no cartilage was observed in the modiolus at any of the fetal stages analyzed. The ossification of the modiolus develops progressively (*μ*CT images in Fig. 4C to H, quantification of the amount of modiolar bone, BV/TV, Fig. 5E), and only small profiles of mineralized bone matrix are visible in histological sections in early stages (Fig. 4D, 27 weeks, upper inset, double arrow). In contrast, the mineral density of the modiolar bone matrix (i.e. its degree of mineralization, TMD) displays an early and fast increase between 24 and 31 wks, when it reaches a plateau, while the pace of ossification accelerates (Fig. 5E).

### Semicircular canals

**Figure 6:**
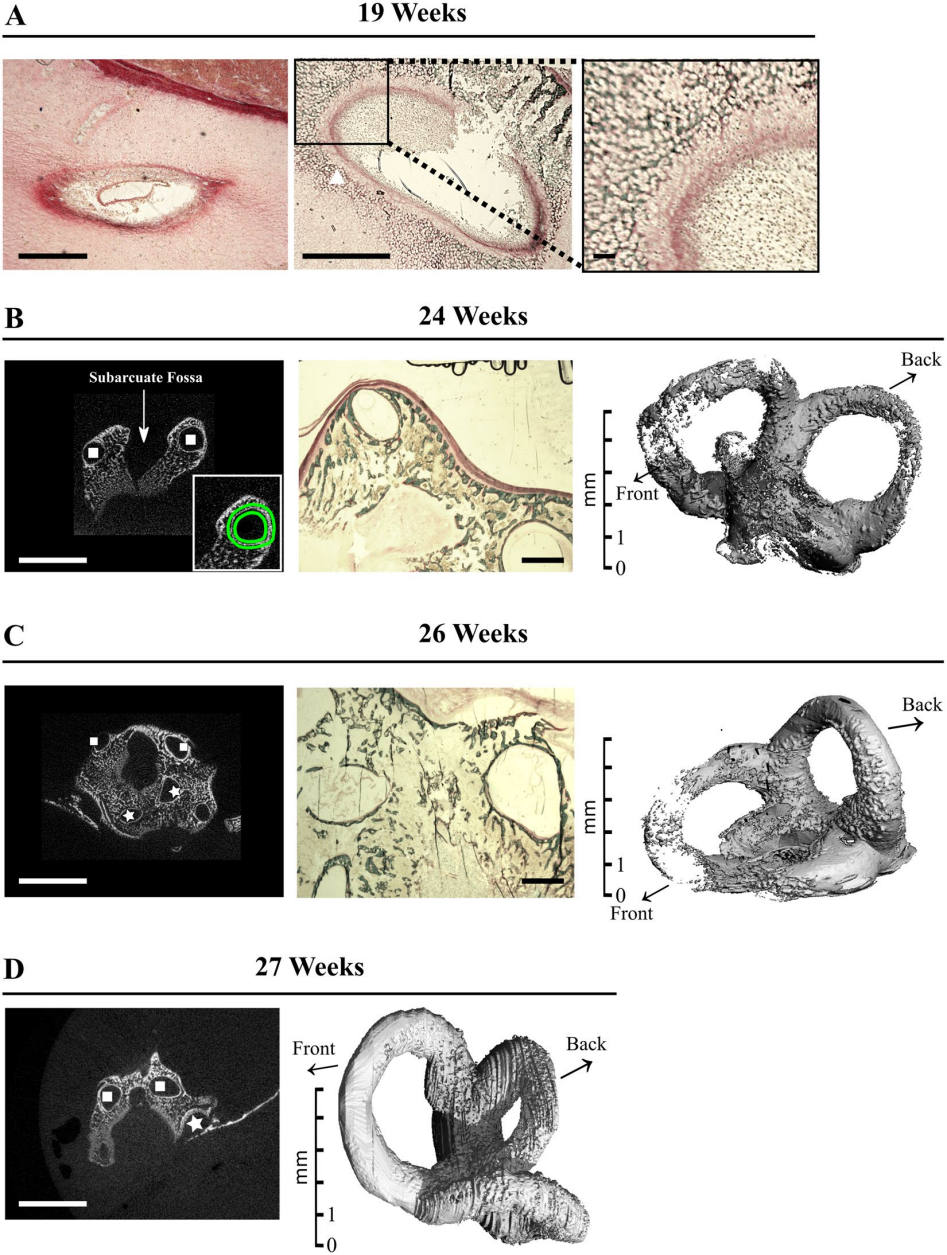
Ossification of the vestibular bony labyrinth. Goldner stained (**A**–**C**) and/or 2D *μ*CT sections and oriented 3D renderings (**B**–**D**), oriented respective to the back and front of the head) of the bony labyrinth between 19 and 27 weeks of gestation. In **A**, Superior Semicircular Canal. Left panel: apical part, Right panels: midportion, with mineralized cartilage (white triangle). Inset in B, left panel: definition of the ROI (green lines) for *μ*CT analysis and 3D rendering of SSC (see Methods for details). In B–D: Stars = Lateral Semicircular Canal, squares = Superior Semicircular Canal. White bars = 5 mm, Black bars = 1 mm, except inset in A: 100 *μ*m.

**Figure 7:**
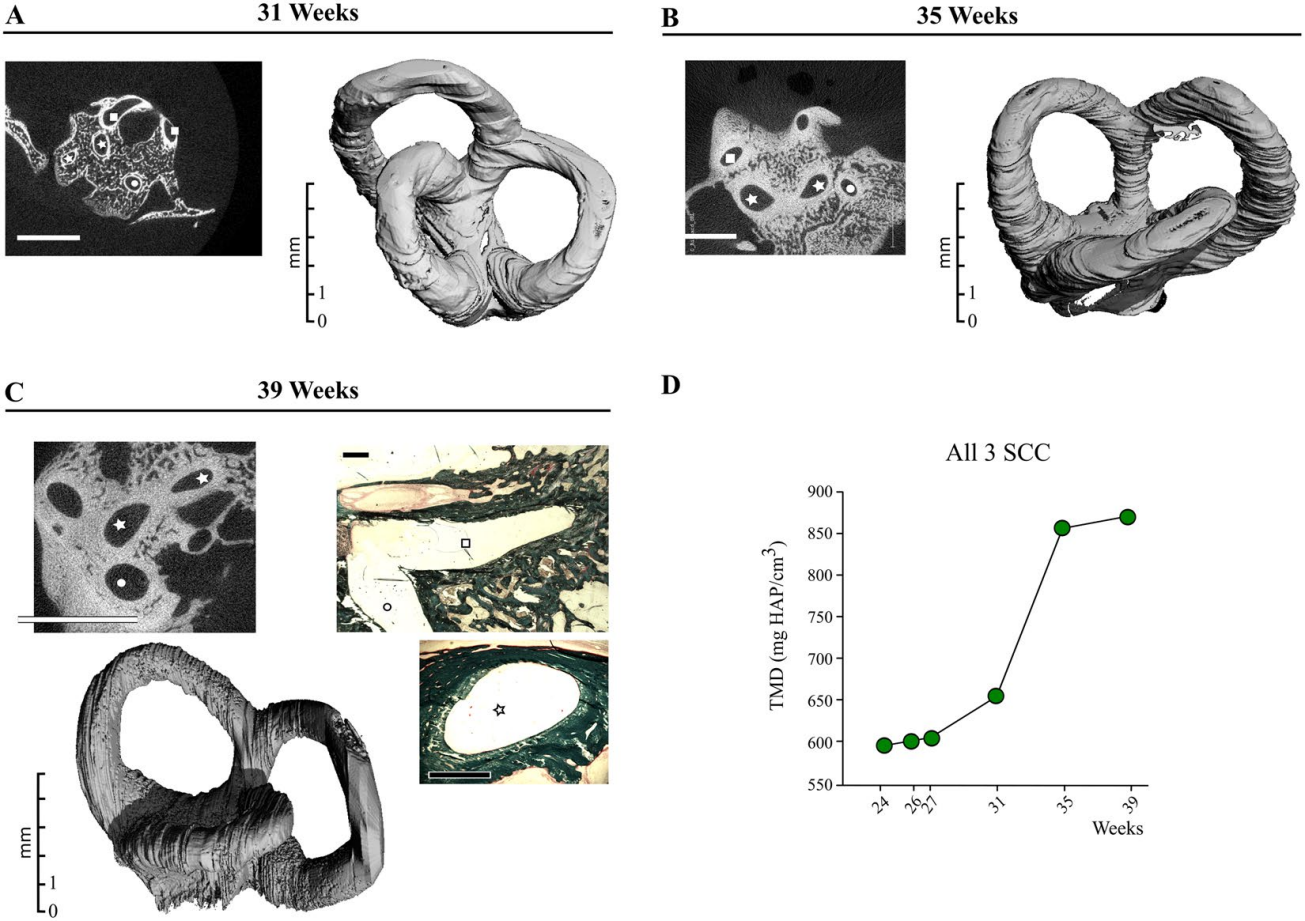
Maturation of the vestibular bony labyrinth. (**A**–**C**) 2D *μ*CT sections, 3D renderings and (**C**) Goldner stained sections of Semicircular Canal (SSC) labyrinth at 31 (**A**), 35 (**B**) and 39 weeks (**C**) of gestation. Stars = Lateral Semicircular Canal, squares = Superior Semicircular Canal, Dot: Posterior Semicircular Canal. White bars = 5 mm, Black bars = 1 mm. (**D**) Time-course of tissue mineral density (TMD) increase in all three SSC (ROI in Fig. 1C) at successive weeks of gestation.

The vestibule is located in the posterior-superior part of the membranous and osseus labyrinth. Similar to the middle ear ossicles and the cochlea, no mineral is detected around the semicircular canals in *μ*CT acquisitions of 17 and 19 wk specimens. At 19 wks, mineralized cartilage is present in histological sections, around the midportion (white square) of the superior semicircular canal (SSCC, Fig. 6A), and not at this stage in its apical part (Fig. 6A), favoring the idea of a basal-apical canal gradient of ossification. Indeed, 3D renderings of the semicircular canals (Figs 6 and 7) show that the basal portion of each canal is the earliest to be mineralized. Considering the bony labyrinth as a whole, bone formation is visible at 24 wks (Fig. 6B), with lacunar external and internal (around the canals) cortices of the otic capsule separated by loose primary trabecular bone (Fig. 6B) still in part cartilaginous (Fig. 6C). The pace of SCC ossification varies also between canals. The SSCC is first seen to achieve complete mineralization in slices (2D images) and 3D *μ*CT reconstructions at 26 wks, whereas the apical part of the posterior (PSCC) and lateral canal (LSCC) is still partly unmineralized at this stage (Fig. 6B–D). Complete bone encapsulation of the three semicircular canals is observed only at 27 wks (Fig. 6D). The thickening of the cortical bone described in other pieces is visible only by day 31, in the inner cortex (surrounding the canals, Fig. 7A) and it progressively obliterates the trabecular area (Fig. 7B) which is much reduced by 39 wks (Fig. 7C). This late, filling up period is concomitant to a sharp increase in TMD (Fig. 7D).

## Discussion

In this study, we analyzed the time-course of middle and inner ear structure ossification/mineralization throughout the second and third parts of fetal period. Because of the age characteristic of the population studied and the prevalence of syndromic stillborn fetuses, it is difficult to obtain a large control group of fetuses devoid of syndroma or middle/inner ear abnormalities, and the small size of the group studied (1 individual per time point) is clearly the main limitation of our study. Nevertheless, this is to the best of our knowledge the first description of human inner ear development integrating high resolution *μ*CT 3D imaging and mineral density measurement with histomorphological analysis of undecalcified skeletal samples. We thus combined accurate 3D imaging (vs. histology sections that may be misaligned using manual reconstruction softwares) at ultra high resolution (higher than MRI^16^) with the high spatial resolution of histology on specimens with preserved bone tissue characteristics (undecalcified). Moreover, we are here providing observational developmental data on a highly sensitive population, and these are of direct interest for the interpretation of clinical symptoms.

Genetics experiments revealed a coordinated morphogenesis of the external and middle ear components^17^. Histological observations^18, 19^ showed a concomitant maturation of anatomical structures with the onset of hearing thereby suggesting an interplayed development of the external auditory canal, the middle ear cavity, inner ear structures and hearing physiology, driven by regulatory mechanisms^17, 18, 20^. Our data are in accordance with previous studies^18, 19^ which reported fully ossified incus and malleus (with the exception of the distal extremity of the malleus handle) by 26 wks. Histomorphological analysis coupled with *μ*CT data allowed us to document the time-course of ossicle ossification and mineralization. In agreement with previous studies^18, 19^. The heads of the malleus and the incus were found to contain bone marrow cavities still visible at 39 wks, which will persist until at least 25 months after birth^21, 22^ in keeping with the possible role of ossicles as blood-forming tissue^21^. The pattern of ossicles ossification could also influence their strength^23, 24^. The progressive filling-up with mineralized matrix is concomitant with the development of the auditory system. This suggests a role for the middle ear ossicles in sound perception by the fetus during the third trimester of pregnancy, even though transmission of skull vibrations to the cochlear fluids has been shown to be the dominant mechanism of sound perception in utero^18, 25, 26^.

The bone mineral density that we measured in the incus at birth (39wks) is far below the values reported from 2 years of age (751 mg HAP/cm3 for the incus, vs 1320 ± 0.07 in 16) thereby indicating further secondary mineralization of middle ear ossicles after birth, also suggested by the temporal dynamics (cf Fig. 3B). This is congruent with a global postnatal maturation of middle ear components^27^. Middle-ear developmental function results not only from development of the ossicules, muscles, cavity and tympanic membrane but also from outer factors related to head size and morpho-histogenesis of the middle ear region, all harmoniously synchronized^28^. A comprehensive description of the close relation between middle-ear closure and the temporomandibular joint (TMJ) has been provided by Rodriguez and colleagues^29–31^. Fetal development of the later and its ligaments is in direct relation to the temporal bone (through its attachment to the tegmen tympani) and its extension with the anterior ligament of the malleus can lead to hearing symptoms in case of TMJ dysfunction^32^. The pneumatization of the middle ear (thought to influence its development^19, 27, 32^ along with that of the tympanic and petrous bones) began during the fetal period and will continue into teenagehood^19^. Pneumatization of the tympanum and epitympanum are completed by birth. However middle ear cavity will continue to enlarge postnatally by expansion of the antral and mastoid air sinuses^33^. The tympanic membrane will undergo structural^34^ and positioning changes^19^. Our observations regarding the incudomallear and incudostapedial joints are in accordance with previous data^18^. Specifically, the development and mobilization of the incudomalleal articulation has been suggested to promote the maturation of the incudostapedial joint^35^ (cf Figs 2B,C and 3C), consistent with a delayed mineralization of the stapes bone (Fig. 3B)^36, 37^. Of note, in the mid-term fetuses the still closed external acoustic meatus and the narrowed tympanic cavity limit joint movements^26^. However a minor mobility of the ossicles has been reported during the late part of the fetal period, mostly related to deglutition^22^.

The ossification of the otic capsule starts about at the same time as that of the ossicles, when these structures reach their adult size^35^. Ossification has been shown to develop from 14 centers merging by 23 wks^38^ to form a bony capsule which has been divided into three distinct layers: the inner cortical bone (inaccurately called “endosteal layer”) lining the perilymphatic space, the trabecular bone area (“Middle Layer”) and the outer cortical bone (incorrectly called “periosteal layer”) as seen on the 2D *μ*CT images (Fig. 4C). Previous works on decalcified specimen described a development of the trabecular bone without thickening of the inner cortex^4^. However, the evolution of techniques allowed us to clearly visualize the maturation of the otic capsule bone, proceeding through a predominant thickening of the external cortex and bone marrow cavity obliteration. Sparse area of persistent calcified cartilage, known as globuli interossei (interosseal globules), are present at birth, in the trabecular middle layer in between the two cortical layers (as we observed, Fig. 4H) whose peculiar changes throughout life play a role in ostosclerosis^39^. Overall, the middle ear ossicles and the cochlear bony labyrinth seem to follow the same pattern of rapidly increasing bone volume through cortical thickening and densification, along with matrix mineralization (Figs 3B and 5E). Although the morphology and morphometry of middle and inner ear at birth are similar to those of adults, our observations indicate a postnatal maturation of the interossicle articulations along with the secondary mineralization of all ear bony structures.

The human fetus responds to sounds after about 21–23 wks^25, 40, 41^ of gestational age, concomitant with a complete bony covering of the cochlea by the inner cortical bone as seen on 2D *μ*CT images. Studies on cochlear hearing physiology have focused more on the relationship between the stiffness of the basilar membrane supporting the organ of Corti and frequency perception than on the bony coverage of the related part of the cochlea. It was shown that the thicker and stiffer the basilar membrane is, the higher the frequency perceived^42^. Our observations of a greater thickening of the basal cornfdqtex/“inner layer” of the cochlear envelope suggest that this feature could play a role in the progressive acquisition of high frequency perception. The fast mineralization of the modiolus is strikingly concomitant to rapid axonal myelination triggered by the onset of hearing (Fig. 5F)^43–45^ and also suggests a functional link. Such hypothesis is supported by previous works on the characteristics of the inner cortex of the bony cochlear labyrinth which were shown to directly influence the functionality of adjacent inner ear structures and consequently affect the hearing^46–48^. Although the adult cochlear size is reached by 23 wks^49^, the still ongoing mineralization of the cochlea at birth time (Fig. 5E) suggests a process of postnatal mineralization, congruent with further hearing development.

Analysis of the ossification of the vestibule provides evidence of a progressive, basal to top process, with a distinct pace for each SSC (Figs 6 and 7). Both histological and *μ*CT data suggest similar pattern for the growth^49^ and the ossification of the 3 canal envelopes, the SSCC bony covering being completed first, followed by the PSCC and then by the LSCC, (Fig. 6). The semicircular canals as well as the cochlea are known to play significant role in vertebrate biology^50^, and under general agreement the bony labyrinth that mirrors the within membranous labyrinthine structures is often used to assess both the form and function of the inner ear in the absence of the membranous ducts. Congruent with previous 2D histological data on decalcified specimens^51^, our observations indicate the appearance of mineralized cartilage areas by the superior semicircular canal at 19 wks rapidly replaced by mineralized bone deposition allowing for complete covering at 27 wks (Fig. 6D). The brainstem structures of the vestibular pathway, such as the lateral vestibular nucleus are known to start functioning between 19 and 21 wks^52^. The completion of all semicircular canals bony covering by 27 wks evidenced in this study, and the concomitant rotation (upside down placement) of most human fetuses into the birth position^53, 54^ suggest a functional link between the two events. Incomplete coverage of the superior semicircular canal (so-called “dehiscent”), leads in mice to a “circling behavior”^55^ and in humans to Minor syndrome, characterized by both hearing loss and sound/pressure induced vertigo^56^. This emphasizes the close relationship between vestibule and cochlear functions.

Our observations show that at 24 wks the cochlea and middle ear ossicules are completely ossified even though they will undergo further maturation during the fetal development and after birth. In contrast, the vestibular part of the labyrinth is fully ossified only at 27 weeks, which coincides with the beginning of the period of fetal rotation. The use of modern bone imaging techniques allowed us to show the relationship between the completion of ossification and the onset of competence of the different structures of the middle and inner ear.

## Methods

### Specimen collection

Temporal bones were collected from 8 fetuses donated by parents to the Department of Anatomy of University Jean Monnet (Saint-Etienne, France). The present study and its related protocol have been approved by the University Hospital Ethics Committee of Saint-Etienne (11.12.2007), the French Data Protection Authority, and the French Biomedicine Agency (number 08.072/02.28.2008). This research was conducted in agreement with the French law pertaining to medical research and written consent was obtained from parents before data collection began. All fetuses deaths occurred as a result of miscarriage or intrauterine death at respectively 17, 19, 24, 26, 27, 31, 35 and 39 estimated gestational weeks (computed to the nearest tenth of a week). In order to outweigh the considerable biological variance in this developmental period, estimates of gestational ages were obtained by completing menstrual age with standard morphometric parameters (maximum length, skull-heel length, biparietal diameter 43). Temporal bones were removed 8 days after death by meticulous dissection to avoid head disfigurement. Fetuses “autopsies did not disclose any cerebrospinal abnormality or syndromic malformation. Family histories showed no congenital anomaly and the fetuses” karyotypes showed no genetic abnormalities.

### High-resolution *μ*CT imaging and analysis

Fetus temporal bones, fixed using neutral buffered 10% formalin and then ethanol-dehydrated were scanned with a high resolution microcomputed tomograph (*μ*CT; VivaCT-40, Scanco Medical, Zürich, Switzerland). All samples were scanned at 45 kVp (176 *μ* A) for 17 and 19 wk fetuses and 55 kVp (142 *μ* A) for others. Exposure time was 4000 ms during which two X ray image projections were acquired at each angular position. Microtomographic scans provided high resolution images with a nominal isotropic voxel size of 10 *μ* m^3^. After reconstruction of 2D slices, Regions Of Interest (ROI) were outlined (see Figs 3, 5 and 6), and 3D reconstructions and volumetric measurement were generated using following parameters: Sigma = 1.4; Support = 3; Lower Threshold = 184; upper threshold = 500. Tissue mineral density (TMD, normalized on an hydroxyapatite (HA) phantom standard and expressed in mg HA/cm^3^) and bone tissue volume/total volume of the ROI (BV/TV, %) parameters were calculated by integration of the linear attenuation coefficient of thresholded bone on each transverse section where the ROI was outlined. For 3D reconstruction of the middle ear, the ROI surrounded each ossicle (example in Fig. 3A). For the cochlear inner cortex and the SCC, the ROI outlined the inner cortical bones (examples in Figs 5D and 6B) until the limit of the round window for the cochlea, and from the apex down to the border of the oval window for the SSC. The TMD of the inner cortical bone was measured on the whole vestibular labyrinth as reconstructed in the 3D renderings (cf Figs 6 and 7). The thickness of the outer and inner cortical bone of the cochlea was measured on twelve 2D slices, manually centered over the section displaying the maximal apparent area of the modiolus. Measurements were performed at 6 pre-defined cortical locations outlined as rectangular windows (see Fig. 5A). These encompassed the highest midpoint of the apical turn, the internal part of the basal turn (midpoint of the basal turn’s lateral wall) and the mid-basal turn on the opposite side (external part, see Fig. 5A). The apical window of the inner cortical bone was also used as an ROI for TMD measurements (cf Fig. 5 orange box). Modiolar TMD and BV/TV were assessed within a ROI encompassing 17 consecutive 2D sections of the modiolus, centered on the slice showing the highest apparent area of the structure. To avoid variability in the visual definition of the limit of the modiolar bone upon the orientation of the specimen during *μ*CT, the ROI was defined for all specimens as a 960 *μ*m^2^ area circle centered on the modiolus.

### Histology of undecalcified bone tissue

Processing of undecalcified bone for histology was as previously described^57^. Briefly, after *μ*CT imaging the samples were embedded in methylmetacrylate at 4 °C, and 9 *μ*m thick sections, parallel to the plane of the superior semi-circular canal were cut with a PolycutS microtome (Leica, Wetzlar, Germany). The sections were stained with modified Goldner’s Masson Trichrome (Goldner), observed under a DMRB optical microscope (Leica, Wetzlar, Germany) and imaged with the Bone Morpho software (Exploranova, La Rochelle, France).

### Data availability statement

Materials, data and associated protocols are promptly available to readers without undue qualifications in material transfer agreements.
